# Effect of a Multidimensional Pharmaceutical Care Intervention on Inhalation Technique in Patients with Asthma and COPD

**DOI:** 10.1155/2020/8572636

**Published:** 2020-11-18

**Authors:** Wanqing Wang, Tao Xu, Qiong Qin, Liyan Miao, Jian'an Bao, Rong Chen

**Affiliations:** Department of Pharmacy, First Affiliated Hospital of Soochow University, Suzhou 215006, Jiangsu, China

## Abstract

**Background:**

Inhalation therapy is the main treatment for asthma and chronic obstructive pulmonary disease (COPD) patients. Owing to the poor inhaler technique in using inhalers, we assessed the effect of a multidimensional pharmaceutical care on inhalation technique in patients with asthma and COPD.

**Materials and Methods:**

A 3-month controlled parallel-group study was undertaken in asthma and COPD patients using dry powder inhalers (DPIs). Patients in the intervention group received multidimensional pharmaceutical care, including establishment of a special dispensing window, face-to-face demonstration and education, brochure education, videos education, online consultation and education, and follow-up reeducation. Patients in the control group received usual pharmaceutical care. The inhaler technique score, correctness of inhaler usage, beliefs about medicines questionnaire (BMQ) score, asthma control test (ACT), and COPD assessment test (CAT) were measured pre- and postintervention. Quality of life improvement evaluated according to score changes of ACT in asthma and CAT in COPD and patient satisfaction were measured postintervention.

**Results:**

259 patients finished the study with 133 in the intervention group and 126 in the control group. Compared to preintervention and control group postintervention, the inhaler technique score, correctness of inhaler usage, and ACT score significantly increased in the intervention group postintervention, while the BMQ score and CAT score decreased significantly (*P* < 0.05). Significant improvements in quality of life and patient satisfaction were found (*P* < 0.05).

**Conclusion:**

This study showed the multidimensional pharmaceutical care for asthma and COPD patients were effective in improving inhalation technique. By providing pharmaceutical care, pharmacists might help asthma and COPD patients to acquire better quality of life.

## 1. Introduction

Respiratory diseases are the main causes of high morbidity and mortality worldwide [1], while COPD has become one of the leading causes of death in China. Inhaled medication is the cornerstone in the treatment of asthma and COPD [2]. Various types of inhaler devices are used [3], including pressure quantitative inhalers (pMDIs), DPIs, and soft mist inhalers (SMIs). The operation steps of these devices are complex, and the essential techniques for each type of device are also different. Hence, it is difficult for patients to operate and master.

Global Initiative for Chronic Obstructive Pulmonary Disease (GOLD), 2017, first describes in detail that errors are frequently made in using inhalers [4]. Studies [1, 5, 6] indicated that incorrect techniques were inevitable in common use of inhalers, which could lead to poor treatment outcome and disease control [5, 7, 8]. In addition, inadequate inhalation technique had been associated with increased emergency department visits [6], additional medical cost, and increased risk of death [6, 9]. It was proposed that incorrect technique attributed to poor adherence to inhaler medication, lack of knowledge, patient's perception of medication, and inadequate education [10–12].

In fact, current education and instruction on inhaler technique are insufficient. A systematic review revealed that about a quarter of patients had never received oral instruction on inhalation technique. Even for those who had received instruction, the quality and time of training and their mastery of inhalation technique were not optimal [13]. Moreover, 86% of patients could not benefit from prescribed drugs due to poor inhaler technique [14]. GOLD 2020 emphasizes that after initial treatment, inhalation technique is an important part of the future review of patients' condition, which cannot be ignored. Therefore, additional interventions are needed for the optimization of education for patients' inhaler technique. The aim of this study was to evaluate a multidimensional pharmaceutical care intervention on inhaler technique improvement in asthma and COPD patients.

## 2. Materials and Methods

### 2.1. Study Population

**Figure 1:**
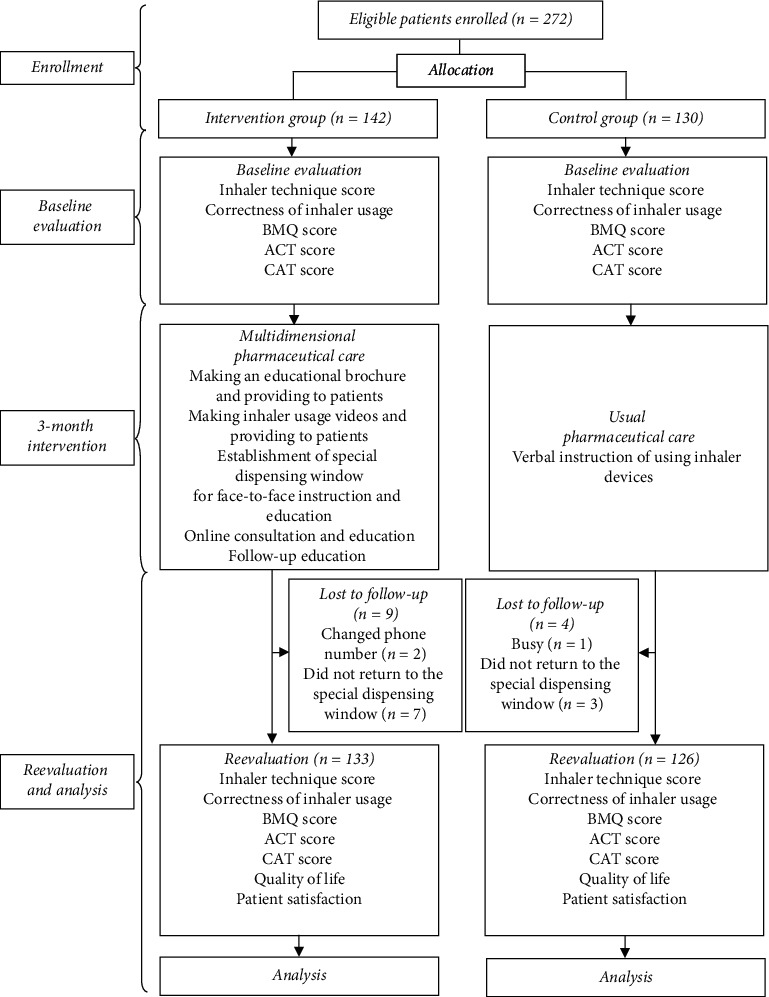
Study flowchart.

A 3-month controlled parallel-group was conducted at the First Affiliated Hospital of Soochow University, Suzhou, Jiangsu, China, from January 2019 to June 2019. Ethical approval was approved by the ethics committee of the First Affiliated Hospital of Soochow University (Ethical approval number: 2014-284), and written informed consent was obtained from all patients. This study also was conducted in accordance with the Declaration of Helsinki in 1964. Patients attended the respiratory department and used one to two of the three DPIs (Turbuhaler®, Diskus®, and Handihaler®) consecutively enrolled. Inclusion criteria were diagnosis of asthma according to Global Initiative for Asthma (GINA) [15] or COPD according to GOLD criteria [4]; using at least one of the three DPIs but without prior experience; could self-administer medicine and had no difficulty in communicating with Chinese; willing to participate; and accept follow-up. Exclusion criteria were patients who had used any one of the three DPIs for more than one week and had been instructed on the three DPIs during the past week. All enrolled patients were under stable condition or experienced only mild to moderate airway obstruction during the study. Study flowchart was shown in Figure 1.

### 2.2. Study Design

A total of 272 patients were enrolled and randomly allocated into an intervention group (*n* = 142) and a control group (*n* = 130). Patients in the intervention group received multidimensional pharmaceutical care intervention, while patients in the control group received usual pharmaceutical care (verbal instruction of inhaler usage). A trained pharmacist collected the baseline information of all patients, including age, gender, educational status, marital status, medical insurance, smoking history, drinking history, diagnosis of diseases, and type of DPIs.

### 2.3. Formulation of a Nine-Step Inhaler Technique

Referring to the manufacture's drug information leaflets and our previous findings on the optimal operation of inhaler technique [16], nine-step inhaler techniques of the three DPIs (Turbuhaler®, Diskus®, and Handihaler®) were developed based on the “seven-step inhaler technique” [17, 18]. The “seven-step inhaler technique” includes opening (device), loading (drug), exhaling, biting (mouthpiece), inhaling, holding breath, and repeating. Considering our previous observations, steps of sitting up before medication and cleaning after medication were added for the optimization of inhaler technique to formulate the “nine-step inhaler technique” (Table 1). Each of the nine steps was scored 1 or 0, by giving 1 for correct operation and 0 for incorrect or missed operation. The total score was 9.

### 2.4. Setting up a Special Dispensing Window for Asthma and COPD Patients

A special dispensing window was established, and three demonstration inhaler devices (Turbuhaler®, Diskus®, and Handihaler®) were provided on this window. Patients with asthma or COPD using one to two of the three devices were automatically allocated to this window and were instructed to operate correct technique by trained pharmacists using demonstration devices in this window.

### 2.5. Face-to-Face Demonstration and Evaluation

Patients were required to demonstrate their inhaler technique using demonstration devices in the special dispensing window. A trained pharmacist observed the process and made an initial assessment (evaluation outcomes included the inhaler technique score, correctness of inhaler usage, BMQ score, ACT score, and CAT score). Patients operating incorrect techniques on each step were checked out and then instructed to perform the correct operation by the trained pharmacist. After that, patients showed the inhaler operation back to pharmacist (teach-back) till no errors were made on every step. Patients could ask questions for further understanding during this process.

### 2.6. Making and Providing an Educational Brochure

An educational brochure was made to improve patient's disease-related knowledge and ability to deal with asthma and COPD. Contents of the brochure include introduction of asthma and COPD diseases, drugs, correct inhaler operation for the three DPIs, quitting smoking, appropriate lifestyle, and the most common problems patients encounter in using inhalers. The brochure was printed and provided to asthma and COPD patients. Patients could watch the brochure multiple times if they needed.

### 2.7. Making and Providing Inhaler Usage Videos

Usage videos of Turbuhaler®, Diskus®, and Handihaler® were taken according to the nine-step inhaler technique and generated into a corresponding two-dimensional code for each inhaler, respectively. Once a patient's prescription was prescribed one of these devices, the drug list delivered to them would automatically generate the corresponding two-dimensional code for the prescribed inhaler. Patients were instructed to scan the two-dimensional code generated on their drug list through the WeChat app (a popular messaging app in China) installed on their mobile phone for watching the usage videos. After leaving the hospital, they could watch the videos at any time. Besides, patients were informed of learning repeatedly till they performed correct technique on all steps.

### 2.8. Establishment of an Online Consulting Platform for Online Consultation and Education

Consultation sections for chronic diseases online had been set up in the WeChat's official account platform of our pharmacy department. One of the sections was opened to asthma and COPD patients. Patients were instructed to scan the two-dimensional code (generated from the WeChat's official account of our pharmacy department) and follow the subscription account. Then, patients could get into the consultation platform through the subscription account and were free to communicate with our pharmacists online. Patients could leave messages on the platform as well. Pharmacists would communicate with them online or responded to their messages. In addition, popular science articles related with asthma and COPD were regularly delivered to patients for learning through the subscription account. Additionally, usage videos of the three inhalers were uploaded to the consulting platform, and patients can watch the videos online at any time.

### 2.9. Follow-Up and Reevaluation

Monthly telephone follow-ups were conducted for three months. During the follow-ups, patients were retrained the inhaler technique and reeducated the correct operation on their operation errors. Patients could ask questions during the process if they had confusion. Patient's assurance of understanding of instruction through telephone was considered as the confirmation of the mastering inhaler technique. After 3 months, patients were invited to the special dispensing window for reevaluation of the inhaler technique by another trained pharmacist (not the pharmacist who conducted the baseline evaluation). Inhaler technique score, correctness of inhaler usage, BMQ score, ACT score, and CAT score were reevaluated. Assessment of the quality of life improvement (changes of the ACT score in asthma and the CAT score in COPD) and patient satisfaction were also conducted.

### 2.10. Evaluation Outcomes

Outcomes were as follows: (1) the inhaler technique score was scored using the nine-step inhaler technique, and the total score was calculated; (2) correctness of inhaler usage was defined as the percentage of patients operating the correct step compared to the total number of patients in the intervention group or control group and evaluated by scoring each step; (3) beliefs about medicines were assessed using the BMQ score [19]. The BMQ was used to evaluate patient's concerns about the harmful results caused by using medicine. Higher score indicated patient perceived a stronger negative attitude to medicine. BMQ was a 9-item questionnaire. The answer “yes” scored 1 and “no” scored 0, and the total score was the sum of all items; (4) quality of life improvement was evaluated according to the changes of the ACT score and CAT score exceeding minimum clinically improvement difference (MCID), which was defined as changes of the ACT score was 3 points [20] and CAT score was 2 points [21]; and (5) patient satisfaction questionnaire was designed according to the previous publication [22]. The satisfaction questionnaire consisted of 8 items. The answer “yes” to items 1–7 scored 1 and “no” scored 0. The item 8 was suggestions proposed to the pharmaceutical care mode by patients.

### 2.11. Data Analysis

Data analysis was conducted using the Statistical Package for Social Science (SPSS 19.0) software (IBM Corp, Armonk, NY, USA). Continuous data were presented as mean ± standard deviation (SD), and categorical data were presented as percentages and frequency. Baseline characteristics of two groups used unpaired *t* tests for continuous data and chi-square tests for categorical data. For the between-group comparisons, Mann–Whitney *U*-tests were performed for continuous data and chi-square tests were used for categorical data. For intragroup comparisons between pre- and postintervention, the paired *t*-test was conducted for continuous data and chi-square tests were used for categorical data. A level of *P* < 0.05 was considered statistically significant.

## 3. Results

### 3.1. Patient Demographic Characteristics

A total of 272 patients were enrolled at baseline, and 259 patients completed the study after a 3-month follow-up (9 lost to follow-up in the intervention group and 4 lost to follow-up in the control group). Patient demographic characteristics are presented in Table 2. The mean age of all patients was 51.20 ± 17.70 years, with 52.90% of males and 47.10% of females. In the participants, 196 patients had asthma and 63 patients had COPD, with 229 patients married and 232 patients who had medical insurance. Most patients had a low education status (122 patients with lower education, and 13 patients had no education). Smoking history was presented in 48 patients, and drinking history was presented in 41 patients. The highest patients were Turbuhaler® users (*n* = 165), followed by Diskus® users (*n* = 44) and HandiHaler® users (*n* = 30). No significant difference in patients' demographic characteristics was found between the intervention group and control group at baseline (*P* > 0.05).

### 3.2. Comparison of Inhaler Technique Score between Two Groups

**Figure 2:**
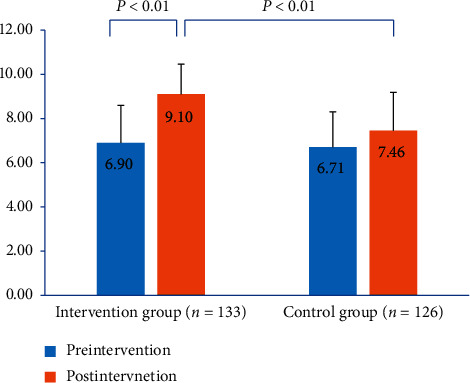
Inhaler technique score in the intervention group and control group pre- and postintervention.

Inhaler technique score was evaluated pre- and postintervention. Significant improvement was found in the intervention group (Figure 2). The increase in the inhaler technique score was significant from preintervention to postintervention in the intervention group (*P* < 0.01). Comparison between the intervention group and control group postintervention was also significantly different (*P* < 0.01).

### 3.3. Comparison of Correctness of Inhaler Usage between Two Groups

The results of correctness of inhaler usage on each step are shown in Table 3. Comparing to preintervention, correctness of inhaler usage in steps 1–5 and steps 7–9 significantly increased in the intervention group postintervention (*P* < 0.05). No significant improvement in step 6 was seen in the intervention group (*P* > 0.05). Significant differences in steps 1–4 and steps 7–9 were found between the intervention group and control group comparison (*P* < 0.05), but no significant changes were found in steps 5-6 (*P* > 0.05).

### 3.4. Comparison of Beliefs about Medicines between Two Groups

**Figure 3:**
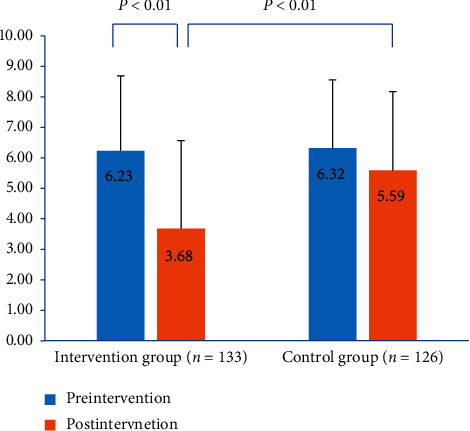
BMQ score in the intervention group and control group pre-and postintervention.

BMQ score in the intervention group significantly decreased postintervention (*P* < 0.01). The change in the BMQ score was also significantly different when comparing the intervention group and control group postintervention (*P* < 0.01, Figure 3).

### 3.5. Comparison of Quality of Life Improvement Pre and Postintervention

The total ACT score was obviously increased, while the total CAT score was decreased significantly in the intervention group from precto postintervention (*P* < 0.05) (Table 4). Moreover, 60.38% of asthma patients and 62.96% of COPD patients reached quality of life improvement exceed MCID in the intervention group. No significant difference in the ACT score and CAT score was found in the control group (*P* > 0.05). What is more, the number of asthma and COPD patients who achieved quality of life improvement exceed MCID was significantly higher in the intervention group compared to the control group (*P* < 0.05 for each).

### 3.6. Comparison of Patient Satisfaction between Two Groups

Patient satisfaction was investigated after 3 months intervention (Table 5). For all the 8 items, patient satisfaction in the intervention group was significantly higher than the control group (*P* < 0.01 of all). In the intervention group, satisfaction in items 1–4 and 6-7 were above 93.23%, but the result of item 5 (77.44%) was slightly lower. The item 8 was patients' suggestions on the multidimensional pharmaceutical care.

## 4. Discussion

This study identified a multidimensional pharmaceutical care based on inhalation technique training and education resulted in a positive effect, and the results showed that the intervention group participants reported better improvements in inhaler technique, correctness of inhaler usage, beliefs about medicines, quality of life, and patient satisfaction postintervention, in comparison to preintervention and control group participants.

GOLD 2020 points out that the effectiveness of inhalation therapy is affected by many factors to varying degrees, including education and training in inhaler technique, medication adherence, and type of operating errors. Education accounts for 90% in the successful treatment of respiratory diseases [1]. Medical staff should not only provide education and training in inhaler usage for patients but also evaluate patients' technique through demonstration and regular follow-up to ensure patients' mastery of correct technique. However, in real life, education in inhaler operation is inadequate in asthma and COPD patients [5]. In this study, a nine-step inhaler technique was formulated based on the seven-step inhaler technique [17] to assist patients in mastering the inhaler technique better and easier. Steps of sitting posture before medication and cleaning after medication were added. Incorrect sitting posture causes a narrow and bent airway, thereby increasing the inhalation resistance which obstructs the natural inhalation of drugs. Furthermore, saliva leaving on the mouthpiece after medication increases the humidity in the mouthpiece and dissolves the powder, therefore reducing the inhaled dose of each suction. According to the operating points of nine-step inhaler technique, we took educational videos of three kinds of DPIs for patients to self-learning at home. Correct inhaler operations were explained in detail and demonstrated by a trained pharmacist in the videos.

In China, with the in-depth development of the medical system reform, pharmacists are facing the transformation from the original guarantee of drug supply to take patients as the center and provide pharmaceutical care for patient. Currently, asthma and COPD are becoming the major challenges for chronic diseases prevention and control in China [17]. Nevertheless, there is no uniform standard for the mode of pharmaceutical care. Patients might perform correct technique on the spot, but their operating techniques dropped off as time went on. Besides, patients might have no other ways of receiving instructions on correct technique apart from the drug information leaflets after leaving hospital [23]. By combining various educational approaches, repeated and continuous education and training on inhalation technique were provided for patients. The information of patients diagnosed with asthma or COPD were automatically allocated to a special dispensing window. Patients were instructed and trained face-to-face by a trained pharmacist in this window. Besides, educational materials including a printed brochure and videos (by scanning the two-dimensional code) were provided to patients. In the brochure, disease-related knowledge and common misunderstandings in using inhalers were illustrated. Also, patients could watch and learn the operation steps in the videos without restriction if they did not remember operations. What is more, patients could consult questions via the consulting platform online or came to the special dispensing window. Telephone follow-up provided an opportunity for pharmacist to reeducate patients in inhaler technique. During the process, individualized pharmaceutical care was performed tailored to patients' needs.

Research studies have showed that inhalation technique can be improved by educational approaches [24, 25]. After education, however, technique skills drop off. Pothirat et al. showed that merely 51% patients had correct technique 1 month after education [24]. According to Blaquiere et al. only 55% patients conducted the correct technique 2 months after training [25]. Distinct improvements in patients' inhalation technique were also identified in our study. The multidimensional pharmaceutical care intervention proved to be effective in improving and retention patients' inhaler technique. Monthly follow-up was conducted in the intervention group participants for repeated education. Moreover, visual materials (a brochure and videos) provided to patients offered a kind of continuous education and instruction, in case their techniques decline over time. Communicating with pharmacists online provided an opportunity for patients to dismiss their confusion timely. Combination of such various educational methods ensured patients mastery of techniques and gave important results. In the intervention group, correctness of almost all steps was above 93% three months after education. The correctness of step 7 (hold breath for more than 5 seconds and optimally for 10 seconds) failed to reach 93%, but the changing degree of this step was the highest (by more than 40%). Patients could not hold breath properly initially. They might make a laugh, speaking, or swallow saliva when holding breath. In addition, the time of patient's holding breath was not long enough. These issues would obviously affect the effective inhalation of drugs. Patients learned how to hold breath correctly and for as long as possible after training and education. It seems that for most patients (above 30%), the major difficult operating steps were step 1 and steps 7–9 (sit up straight and keep the upper part of the body upright, hold breath for more than 5 seconds and optimally for 10 seconds, repeat the fourth to seventh step, clean the mouthpiece, and close the inhaler). The proportions of patients had correct inhaler technique of step 1 and, steps 7–9 were low at baseline. However, after education, correctness in these four steps improved about 20% in the intervention group compared to preintervention and control group. Step 7 was also found to be the difficult step in other studies [1, 26]. The results indicated that education on these difficult steps should be addressed. At every follow-up, repeated instructions and education on the correct use of these steps were conducted by the pharmacist, and therefore, obvious improvements in correct operation were observed. No significant differences in correctness of step 5 and step 6 were found between groups postintervention. This is probably due to the high correctness of the two steps at baseline.

Patients worried that they would indulge in drugs, and their lives would be disturbed by using drugs, owing to long-term treatment of asthma and COPD [27]. Kovaćev ić et al. [27] concluded that increased awareness of association between the future health and nondeterioration could lead to an effective trend by using asthma medications. Analyzing the beliefs about medications in the participants, patients' attitude towards drugs changed evidently, and mean BMQ scores significantly decreased postintervention. This determined that the performance of multidimensional pharmaceutical care gave effective outcomes. Potential negative beliefs about drugs were decreased, and patients began to realize the benefits of using drugs and regular treatment. Inhalation technique is one of the crucial factors for effective asthma management [28, 29]. The improvement of the inhalation technique might contribute to better quality of life. In the present study, besides enhancement of inhaler technique, patient's quality of life improved positively based on our data. In the intervention group, proportions of improvement exceeding MCID were identified clearly higher in both asthma and COPD patients compared to the control group. Although the lung function was not detected in our study, which has been associated with the score changes of ACT for asthma and CAT for COPD. Changes of the ACT score and CAT score probably related with diminishing lung function decline. All the results showed that multidimensional pharmaceutical care based on inhaler technique education and training positively influenced patient beliefs about medicines, inhaler technique, and quality of life.

Patient satisfaction reflects that the overlap of a patient's experience correspond with their expectations. Satisfied patients are more likely adhere to disease therapy, thereby having better outcomes [30]. Patients do need pharmaceutical care provided by pharmacists [22]. We investigated patient satisfaction with the multidimensional pharmaceutical care. Satisfaction was nearly above 93.23% in items 1–4 and items 6-7 postintervention, indicating that patients had high acceptance and satisfaction with the pharmaceutical care. Patients' suggestions on the pharmaceutical care (item 8) mainly focused on the type of diseases and inhaler devices and form, frequency, and coverage of pharmaceutical care. These results denoted that patients made a positive response to the satisfaction survey and had a strong desire for continuous pharmaceutical care provided by pharmacists. Satisfaction of item 5 (77.44%) was lower than other items. This probably arose from the fact that pharmacists primarily focused on education on the inhaler technique skills, while education on disease of asthma and COPD and drug-related knowledge were insufficient. This finding underlined the necessity of optimal individualized pharmaceutical care to meet the needs of more patients.

There were some limitations in this study. First, since the primary objective of this study was to determine the application and practice of the multidimensional pharmaceutical care, we have not conducted the pharmacoeconomic evaluation. Second, as the inhaler devices of patients used in this study were DPIs, data on pMDIs and SMIs are lacking. Further research studies are needed to identify these issues.

## 5. Conclusion

In conclusion, correct inhalation technique is crucial for the successful treatment of asthma and COPD. We underlined the need of multidimensional pharmaceutical care intervention to educate and train patients repeatedly and continuously, and we eventually improved inhalation technique of patients. It is a beneficial attempt to provide active and continuous pharmaceutical care for asthma and COPD patients by the pharmacist in China.

## Figures and Tables

**Table 1:** Nine-step inhalation technique.

	Summary	Nine steps of inhalation technique

Turbuhaler®	Sitting	Sit up straight and keep the upper part of the body upright
	Opening	Open the device by removing the cap from the inhaler
	Loading	Hold the inhaler upright, rotate grip anticlockwise, and then back until a click is heard
	Exhaling	Exhale to residual volume and away from device
	Biting	Place the mouthpiece gently between the teeth and seal the lips round the mouthpiece
	Inhaling	Inhale forcefully and deeply as fast as you can
	Holding	Hold breath for more than 5 seconds (optimally for 10 seconds)
	Repeating	Repeat the fourth step to the seventh step to ensure to get the full dose
	Cleaning	Clean the place where the mouth is bitten on the device and close the inhaler
Diskus®	Sitting	Sit up straight and keep the upper part of the body upright
	Opening	Open the inhaler by pushing the rod
	Loading	Push lever back completely
	Exhaling	Exhale away from mouthpiece
	Biting	Place the mouthpiece gently between the teeth and seal the lips round the mouthpiece
	Inhaling	Inhale forcefully and deeply as fast as you can
	Holding	Hold breath for more than 5 seconds (optimally for 10 seconds)
	Repeating	Repeat the fourth step to the seventh step to ensure get the full dose
	Cleaning	Clean the place where the mouth is bitten on the device and close inhaler
Handihaler®	Sitting	Sit up straight and keep the upper part of the body upright
	Opening	Open the inhaler by pulling the dust cap upwards, and open the mouthpiece
	Loading	Place the capsule in the central compartment and close the mouthpiece firmly
	Exhaling	Exhale away from mouthpiece
	Biting	Place the mouthpiece gently between the teeth and seal the lips round the mouthpiece
	Inhaling	Inhale forcefully and deeply as fast as you can, and a rattle can be heard
	Holding	Hold breath for more than 5 seconds (optimally for 10 seconds)
	Repeating	Repeat the fourth step to the seventh step to ensure to get the full dose
	Cleaning	Clean the place where the mouth is bitten on the device and close the inhaler

**Table 2:** Demographic characteristics of asthma and COPD patients.

Characteristics	Intervention group (*n* = 133)	Control group (*n* = 126)	All (*n* = 259)	*P*

Age, years, mean ± SD	51.01 ± 17.30	51.40 ± 18.19	51.20 ± 17.70	0.860^a^
Gender, *n* (%)				0.736^a^
Male	69 (51.88)	68 (53.97)	137 (52.90)	
Female	64 (48.12)	58 (46.03)	122 (47.10)	
Level of education, *n*				0.461^a^
No education	5	8	13	
Lower education	61	61	122	
Secondary education	25	27	52	
Higher education	42	30	72	
Marital status, *n*				0.186^a^
Married	121	108	229	
Unmarried/divorced/solitary	12	18	30	
Medical insurance, *n*				0.644^a^
Yes	118	114	232	
No	15	12	27	
Smoking history, *n*				0.622^a^
Ever	22	26	48	
Never	101	89	190	
Quit smoking for ≥1 year	10	11	21	
Drinking history, *n*				0.507^a^
Yes	23	18	41	
No	110	108	218	
Diagnosis, *n*				0.121^a^
Asthma	106	90	196	
COPD	27	36	63	
Type of inhalers, *n*				0.554^a^
Tubuhaler®	85	80	165	
Diskus®	26	18	44	
HandiHaler®	13	17	30	
Diskus® and HandiHaler®	4	7	11	
Diskus® and Tubuhaler®	2	3	5	
HandiHaler® and Tubuhaler®	3	1	4	

^a^Comparison between the intervention group and control group postintervention.

**Table 3:** Correctness of inhaler usage score in the intervention group and control group pre- and postintervention.

Time of evaluation	Intervention group (*n* = 133)	Control group (*n* = 126)	*P*

Step 1, *n* (%)			
Preintervention	89 (68.42)	82 (65.08)	<0.001^a^
Postintervention	130 (97.74)	85 (67.46)	<0.001^b^
Step 2, *n* (%)			
Preintervention	94 (70.68)	93 (73.81)	<0.001^a^
Postintervention	127 (95.49)	104 (82.54)	0.001^b^
Step 3, *n* (%)			
Preintervention	112 (84.21)	109 (86.51)	<0.001^a^
Postintervention	130 (97.74)	113 (89.68)	0.015^b^
Step 4, *n* (%)			
Preintervention	97 (72.93)	83 (65.87)	<0.001^a^
Postintervention	129 (96.99)	88 (69.84)	<0.001^b^
Step 5, *n* (%)			
Preintervention	120 (90.22)	120 (95.24)	0.003^a^
Postintervention	132 (99.25)	122 (96.83)	0.335^b^
Step 6, *n* (%)			
Preintervention	120 (90.22)	110 (87.30)	0.087^a^
Postintervention	127 (95.49)	111 (88.10)	0.051^b^
Step 7, *n* (%)			
Preintervention	61 (45.86)	56 (44.44)	<0.001^a^
Postintervention	115 (86.47)	58 (46.03)	<0.001^b^
Step 8, *n* (%)			
Preintervention	79 (59.40)	76 (60.32)	<0.001^a^
Postintervention	124 (93.23)	81 (64.29)	<0.001^b^
Step 9, *n* (%)			
Preintervention	86 (64.66)	82 (65.08)	<0.001^a^
Postintervention	126 (94.74)	84 (66.67)	<0.001^b^

^a^Comparison between pre- and postintervention in the intervention group. ^b^Comparison between the intervention group and control group postintervention.

**Table 4:** Quality of life in the intervention group and control group pre- and postintervention.

Group	Preintervention, mean ± SD	Postintervention, mean ± SD	Post-preintervention, mean ± SD	*P*	Improving exceed MCID, *n* (%)	*P*

Intervention group						
ACT score (*n* = 106)	17.44 ± 4.35	20.94 ± 4.66	3.50 ± 4.65	<0.01^a^	64 (60.38)	<0.01^b^
CAT score (*n* = 27)	19.81 ± 6.74	15.67 ± 6.03	-4.15 ± 5.67	0.021^a^	17 (62.96)	<0.01^b^
Control group						
ACT score (*n* = 90)	17.63 ± 4.37	18.32 ± 4.97	0.69 ± 3.05	0.325^a^	13 (14.44)	
CAT score (*n* = 36)	19.39 ± 5.21	18.44 ± 5.61	-0.94 ± 5.24	0.461^a^	5 (13.89)	

^a^Comparison of the ACT score and CAT score between pre- and postintervention, respectively. ^b^Comparison of quality of life improving exceed MCID between the intervention group and control group.

**Table 5:** Patient satisfaction in the intervention group and control group postintervention.

Items	Intervention group (*n* = 133), *n* (%)	Control group (*n* = 126), *n* (%)	*P*

1 I have not received such a service from outpatient department before	129 (96.99)	73 (57.93)	<0.001^a^
2 I am satisfied with the pharmaceutical care services given by the pharmacist	131 (98.50)	57 (45.24)	<0.001^a^
3 I feel comfortable to communicate with the pharmacist	130 (97.74)	62 (49.21)	<0.001^a^
4 I am satisfied with the pharmacist answered questions I have about my medications	129 (96.99)	57 (45.24)	<0.001^a^
5 My knowledge about disease and medications improved after receiving the services	104 (77.44)	36 (28.57)	<0.001^a^
6 I will recommend this service to my friends and family members who are using inhaler devices	130 (97.74)	50 (39.68)	<0.001^a^
7 I want to receive such a pharmaceutical care service for other diseases	124 (93.23)	62 (49.21)	<0.001^a^
8 I have some suggestions about the pharmaceutical care services	85 (63.91)	33 (26.19)	<0.001^a^

^a^Comparison between the intervention group and control group.

## Data Availability

The data used to support the findings of this study are included within the article.
